# Supplementary figures and images for: The inner nuclear membrane protein NEMP1 supports nuclear envelope openings and enucleation of erythroblasts
Source: PLoS Biol. 2022 Oct 10;20(10):e3001811. doi: 10.1371/journal.pbio.3001811 (PMC9595564; doi:10.1371/journal.pbio.3001811)

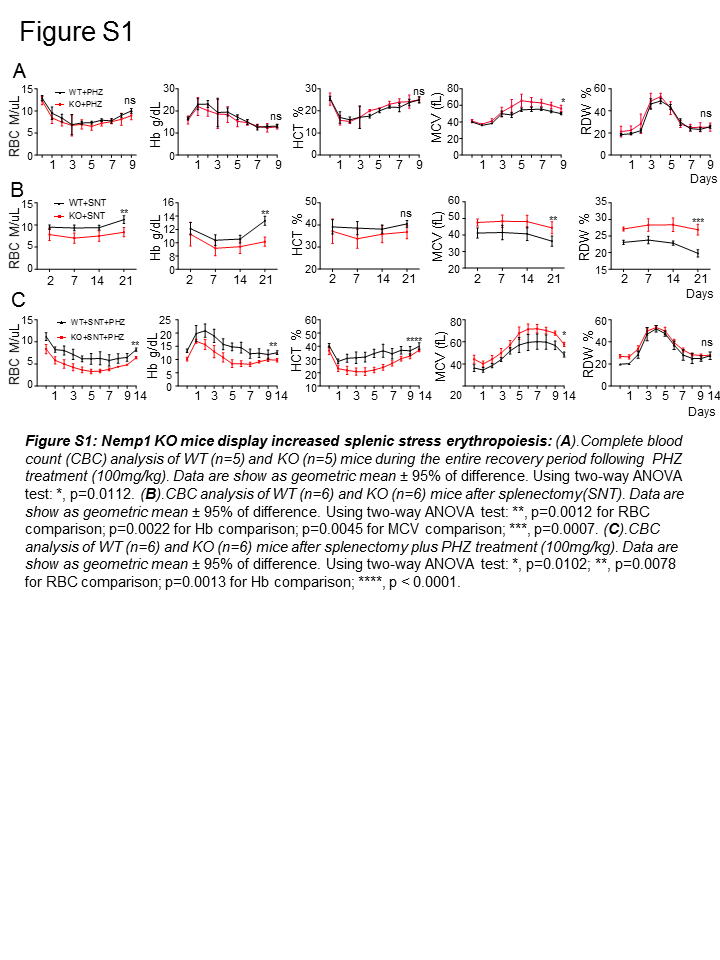

Supplement: S1 Fig — (A) CBC analysis of WT (n = 5) and KO (n = 5) mice during the entire recovery period following PHZ treatment (100 mg/kg). Data are shown as geometric mean ± 95% of difference. Using 2-way ANOVA test: *, p = 0.0112. (B) CBC analysis of WT (n = 6) and KO (n = 6) mice after splenectomy (SNT). Data are shown as geometric mean ± 95% of difference. Using 2-way ANOVA test: **, p = 0.0012 for RBC comparison; p = 0.0022 for Hb comparison; p = 0.0045 for MCV comparison; ***, p = 0.0007. (C) CBC analysis of WT (n = 6) and KO (n = 6) mice after splenectomy plus PHZ treatment (100 mg/kg). Data are shown as geometric mean ± 95% of difference. Using 2-way ANOVA test: *, p = 0.0102; **, p = 0.0078 for RBC comparison; p = 0.0013 for Hb comparison; ****, p < 0.0001. Data underlying the graphs shown can be found in S5 Data. CBC, complete blood count; Hb, hemoglobin; KO, knockout; MCV, mean corpuscular volume; PHZ, phenylhydrazine; RBC, red blood cell; WT, wild type. (TIF) [file pbio.3001811.s001.tif]

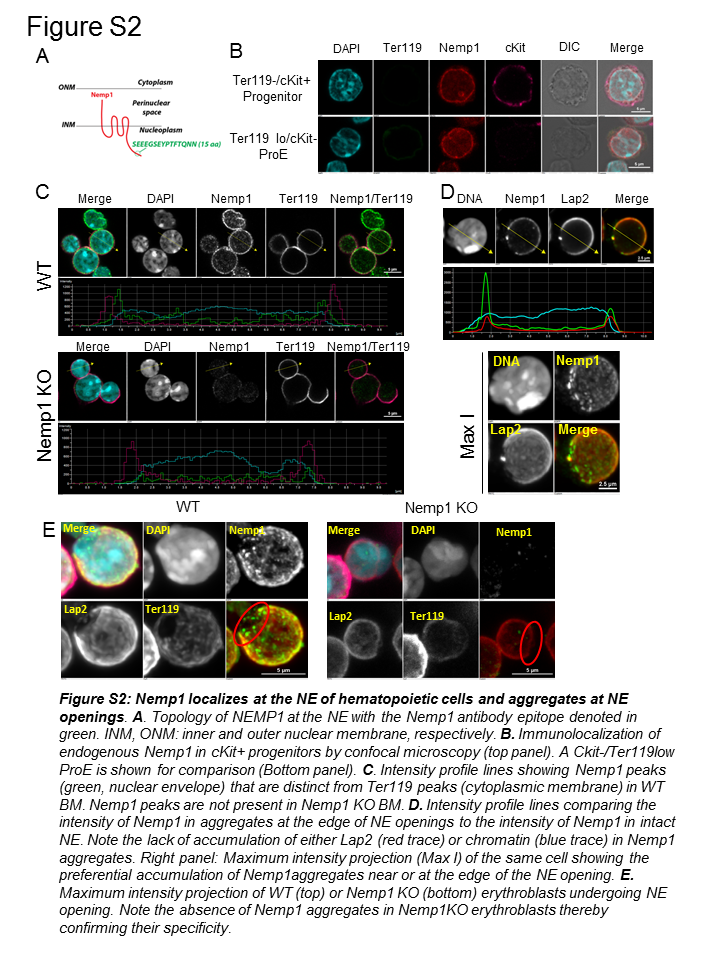

Supplement: S2 Fig — (A) Topology of NEMP1 at the NE with the Nemp1 antibody epitope denoted in green. (B) Immunolocalization of endogenous Nemp1 in cKit+ progenitors by confocal microscopy (top panel). A Ckit-/Ter119low ProE is shown for comparison (bottom panel). (C) Intensity profile lines showing Nemp1 peaks (green, nuclear envelope) that are distinct from Ter119 peaks (cytoplasmic membrane) in WT BM. Nemp1 peaks are not present in Nemp1 KO BM. (D) Intensity profile lines comparing the intensity of Nemp1 in aggregates at the edge of NE openings to the intensity of Nemp1 in intact NE. Note the lack of accumulation of either Lap2 (red trace) or chromatin (blue trace) in Nemp1 aggregates. Right panel: Maximum intensity projection (Max I) of the same cell showing the preferential accumulation of Nemp1aggregates near or at the edge of the NE opening. (E) Maximum intensity projection of WT (top) or Nemp1 KO (bottom) erythroblasts undergoing NE opening. Note the absence of Nemp1 aggregates in Nemp1 KO erythroblasts thereby confirming their specificity. BM, bone marrow; INM, inner nuclear membrane; KO, knockout; NE, nuclear envelope; ONM, outer nuclear membrane; WT, wild type. (TIF) [file pbio.3001811.s002.tif]

## Slide 1
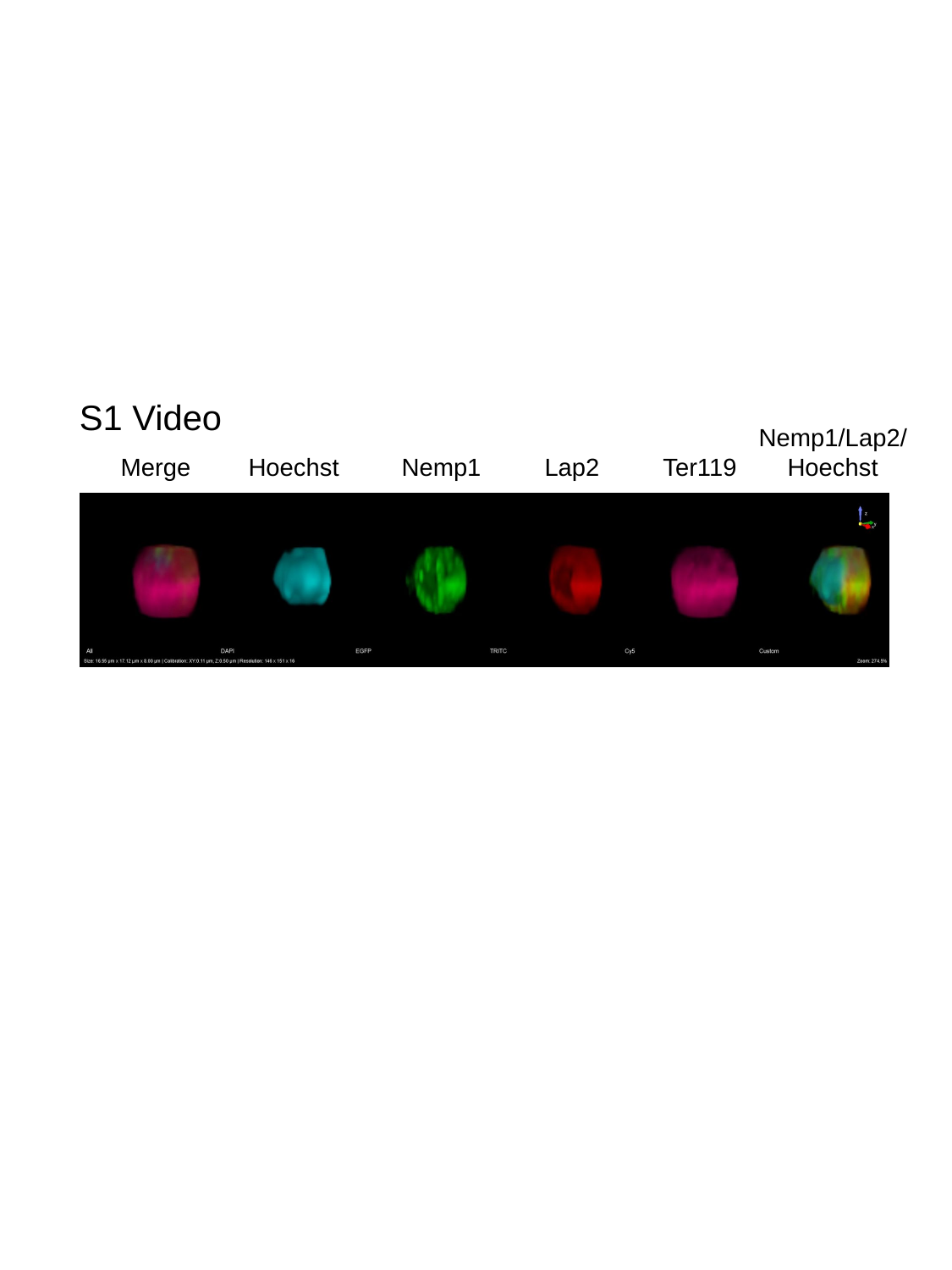

S1 Video
Nemp1/Lap2/
Hoechst
Merge
Hoechst
Nemp1
Lap2
Ter119

Supplement: S1 Video — (PPTX) [file pbio.3001811.s003.pptx]

## Slide 1
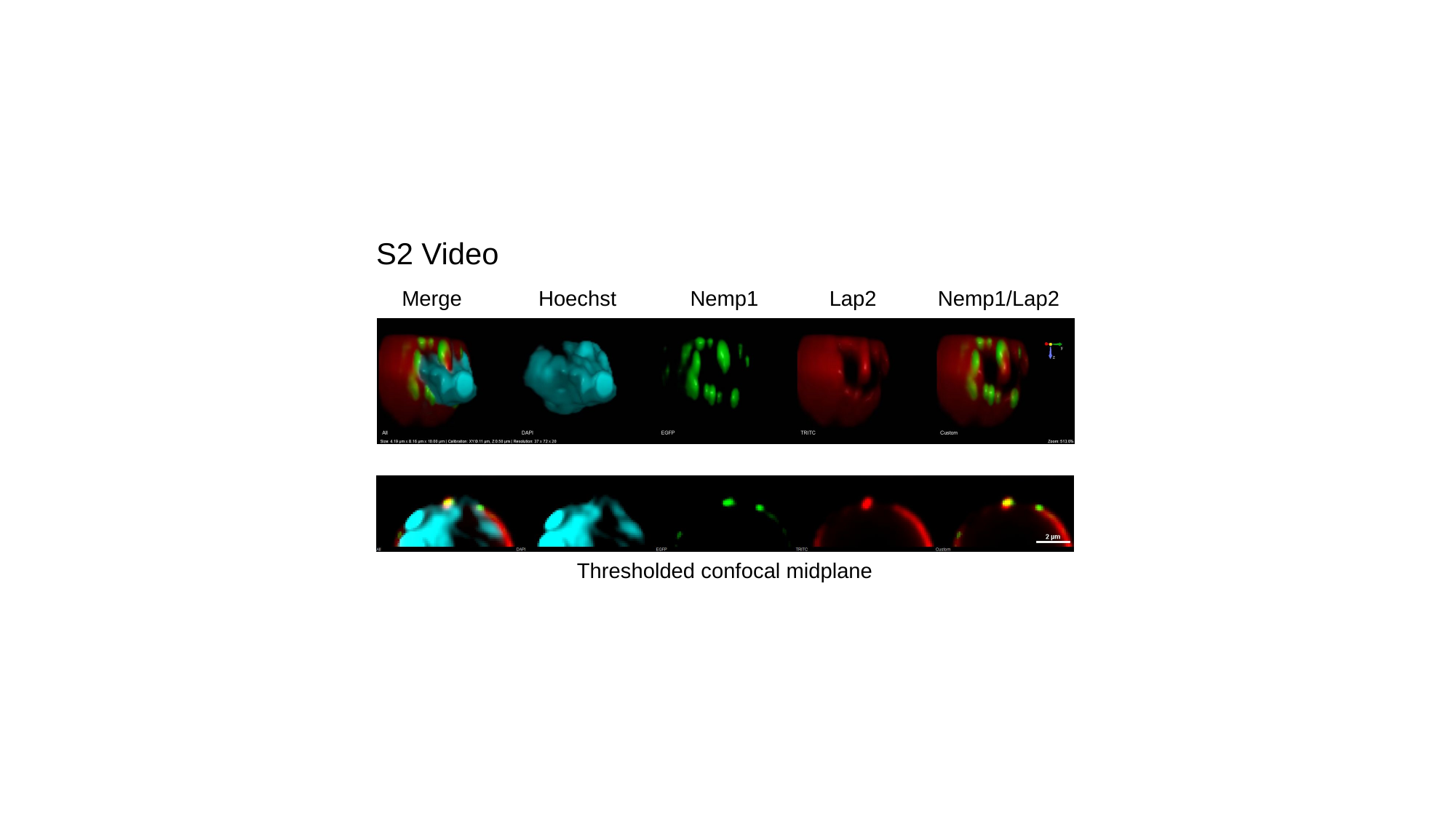

S2 Video
Merge
Hoechst
Nemp1
Lap2
Nemp1/Lap2
Thresholded confocal midplane

Supplement: S2 Video — Note the presence of Nemp1 aggregates near or close to NE openings. (PPTX) [file pbio.3001811.s004.pptx]

## Slide 1
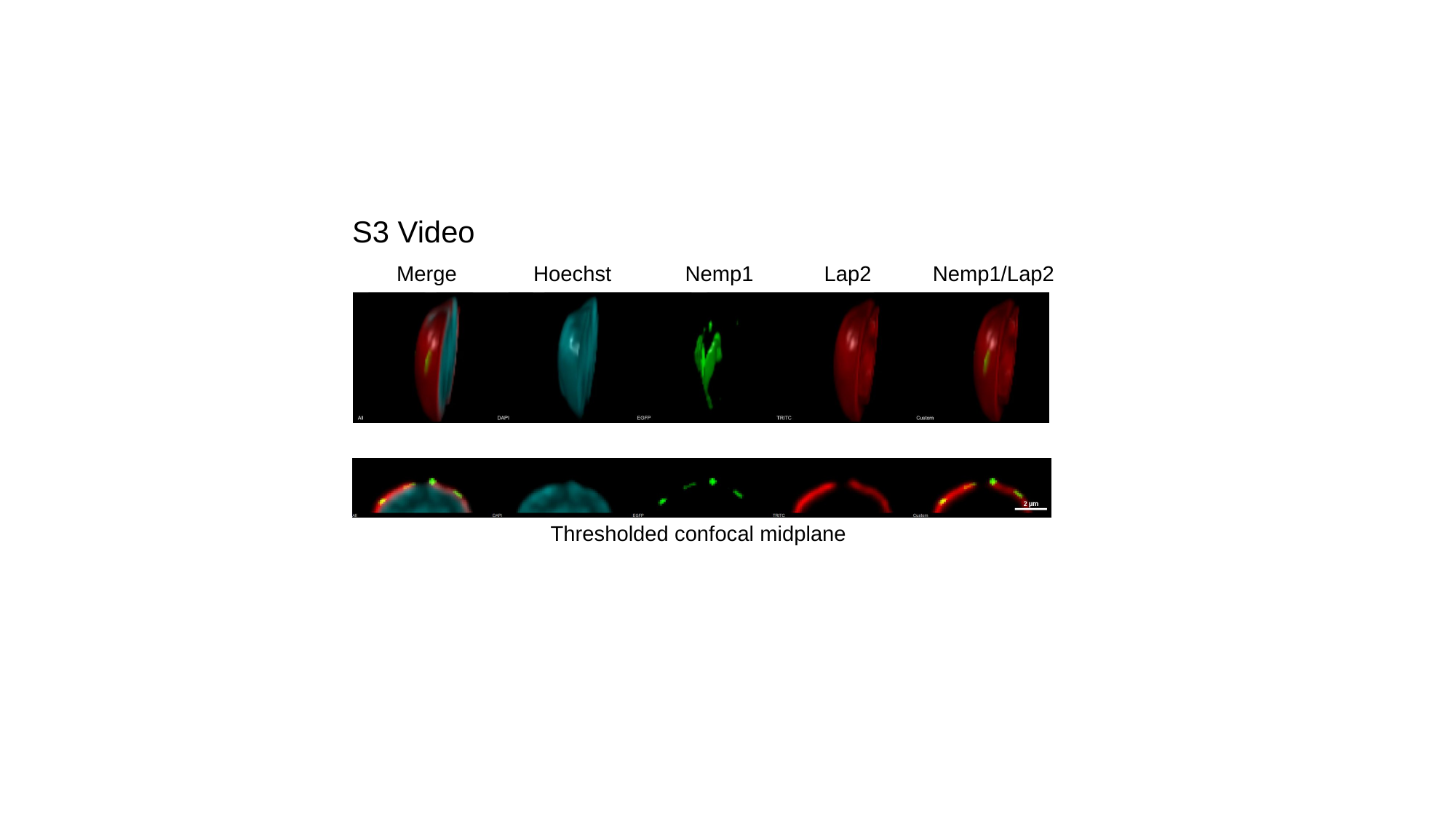

S3 Video
Merge
Hoechst
Nemp1
Lap2
Nemp1/Lap2
Thresholded confocal midplane

Supplement: S3 Video — Note the presence of Nemp1 aggregates near or close to NE openings. (PPTX) [file pbio.3001811.s005.pptx]

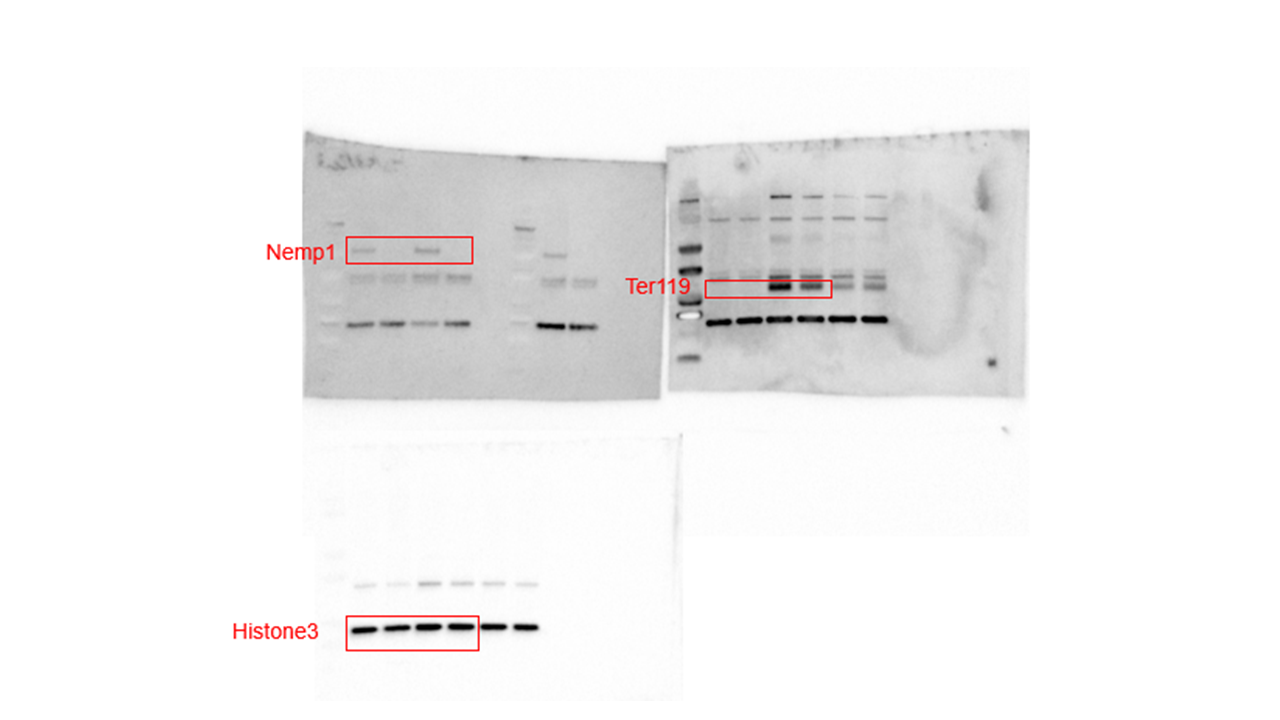

Supplement: S1 Raw image — (TIF) [file pbio.3001811.s011.tif]

WT

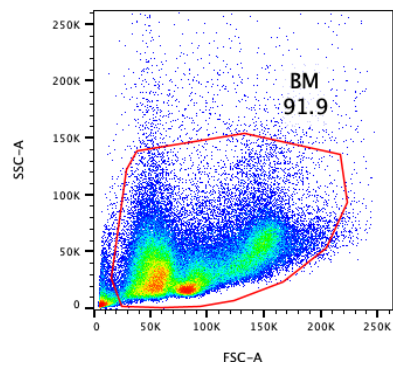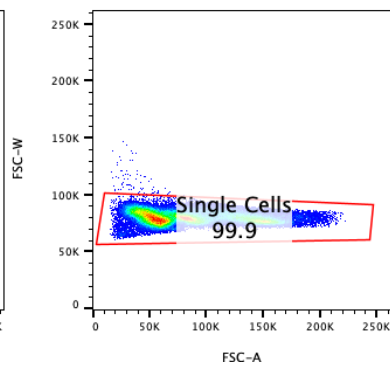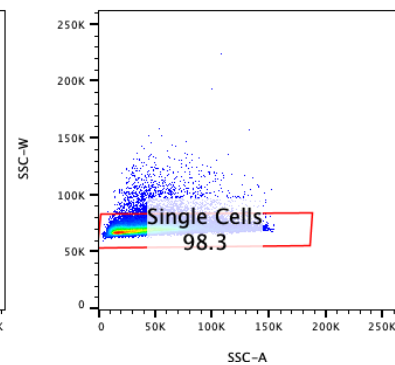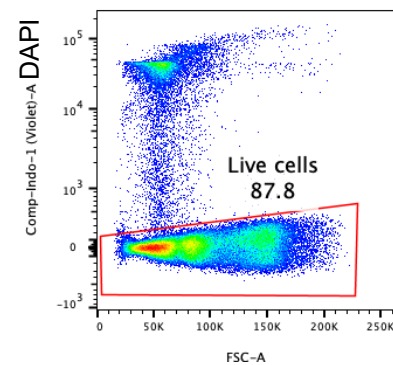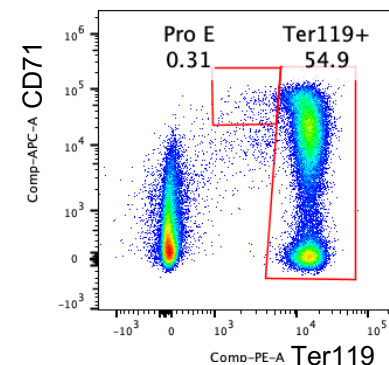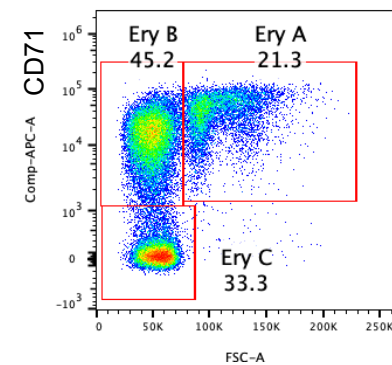

KO

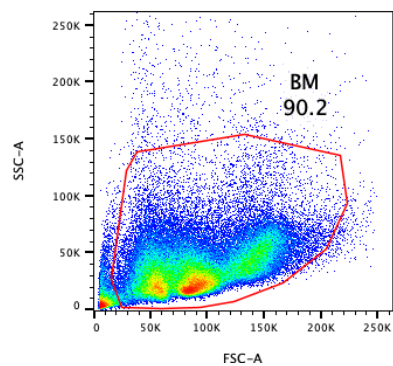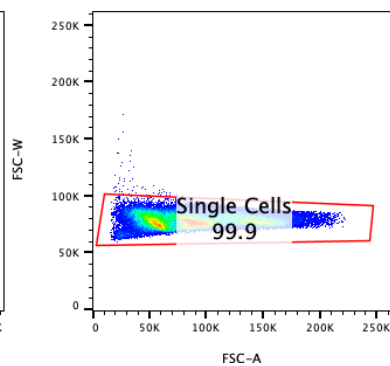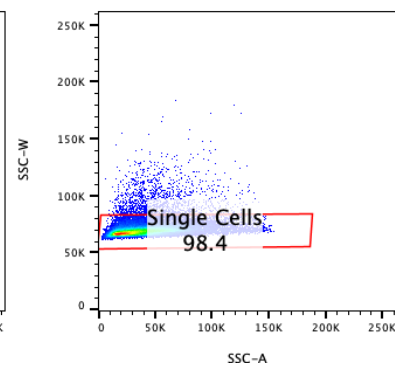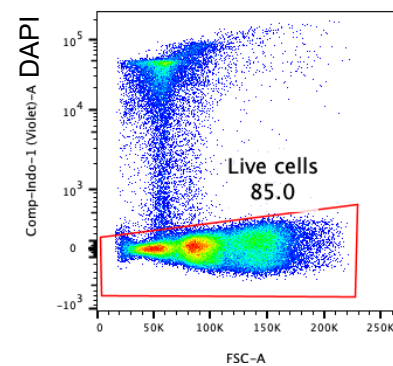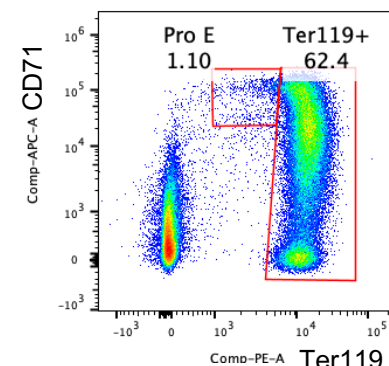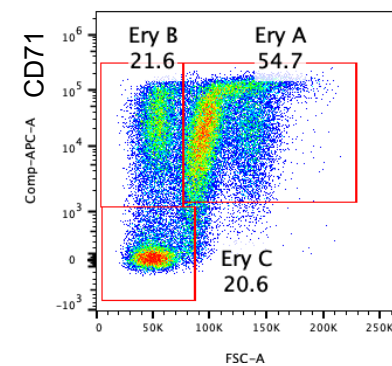

Supplement: S1 Raw data — (ZIP) [file pbio.3001811.s013.zip › S1_Raw_data/Fig 2F.pdf]

WT

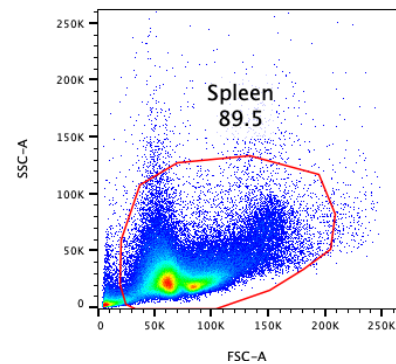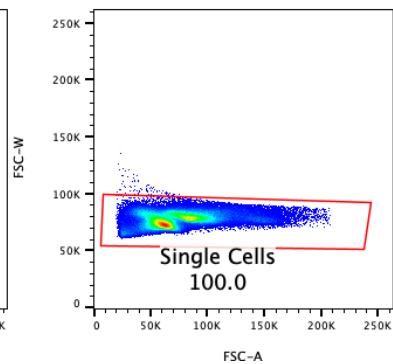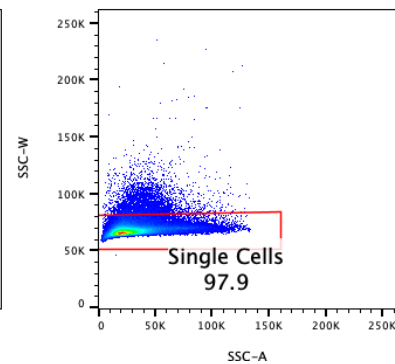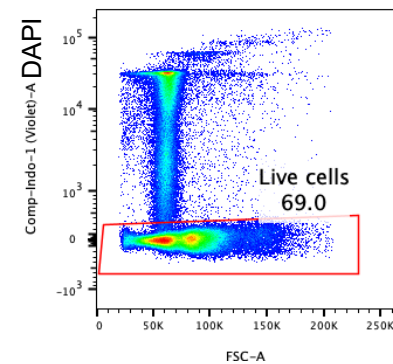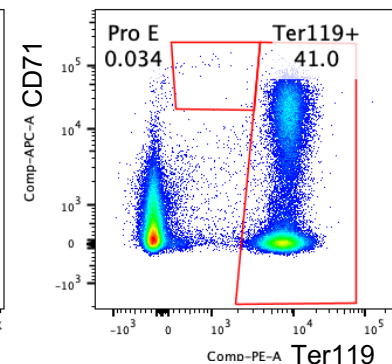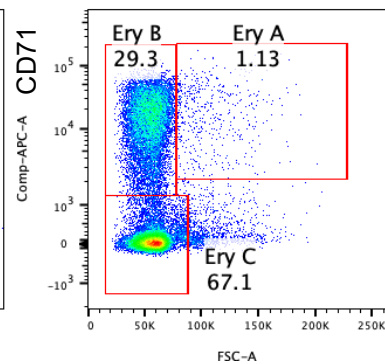

KO

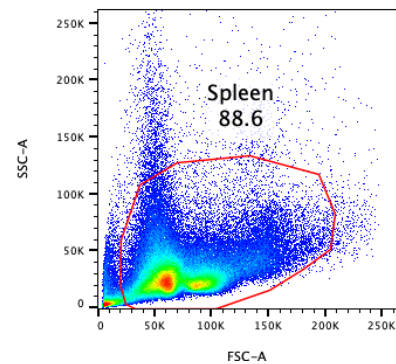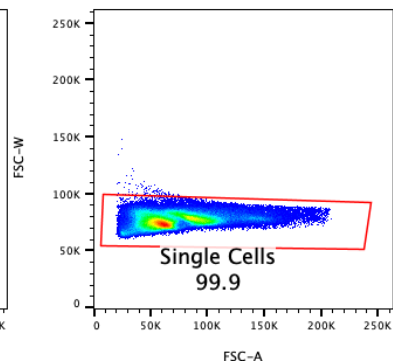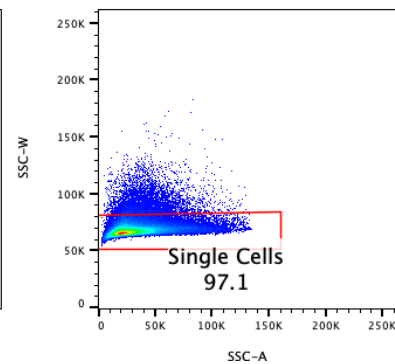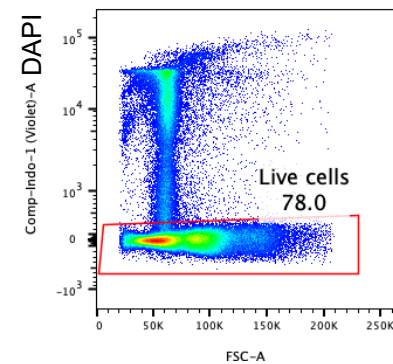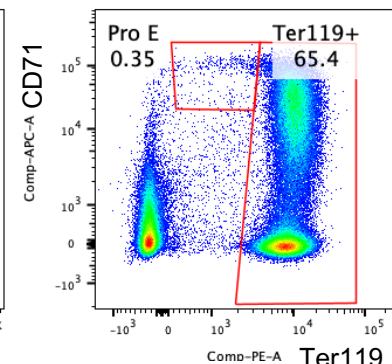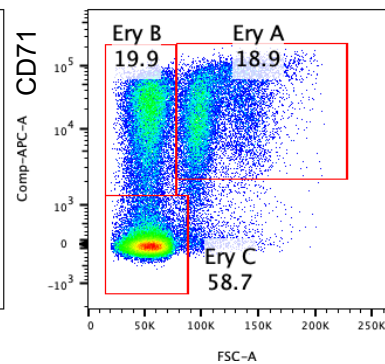

Supplement: S1 Raw data — (ZIP) [file pbio.3001811.s013.zip › S1_Raw_data/Fig 2I.pdf]

WT

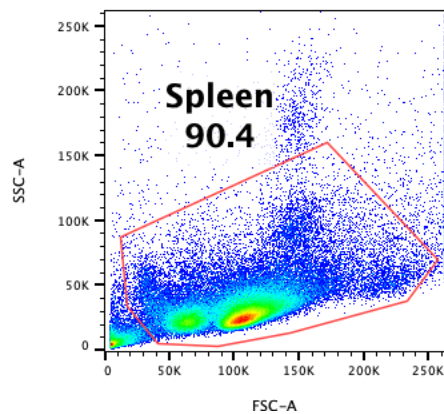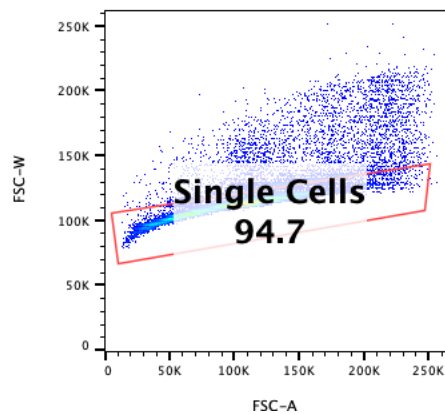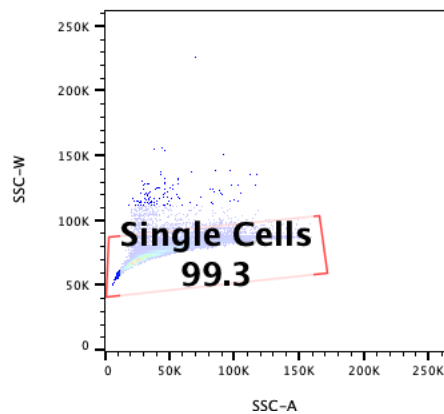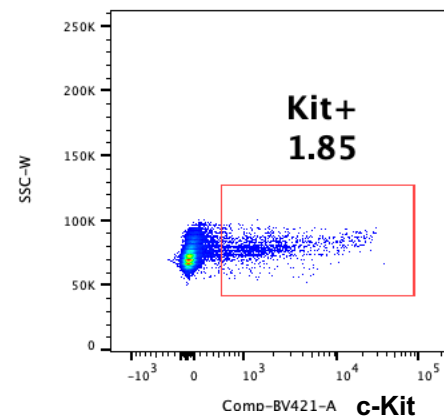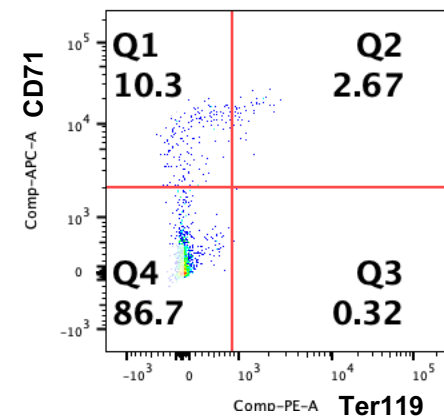

KO

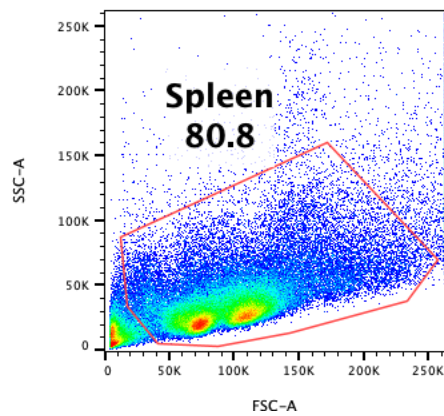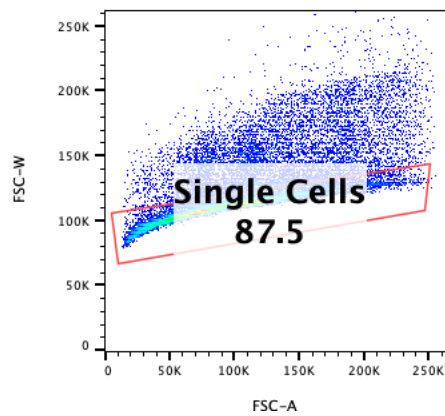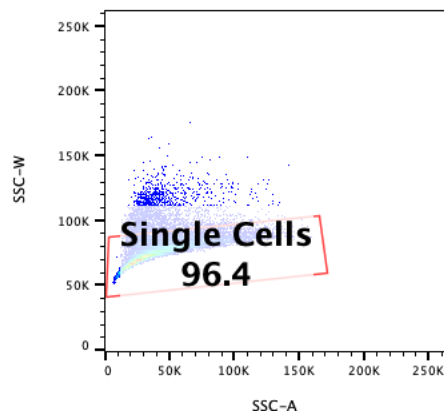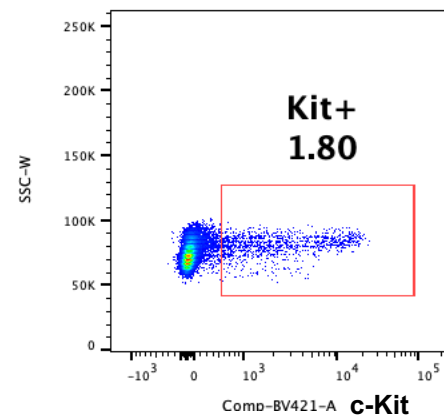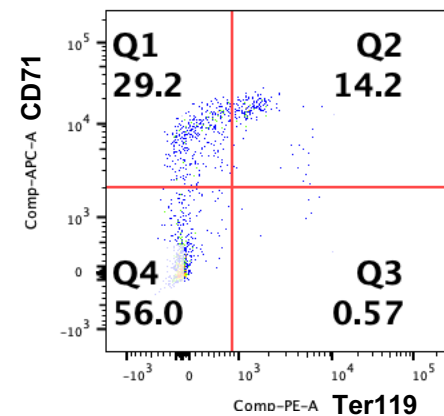

Supplement: S1 Raw data — (ZIP) [file pbio.3001811.s013.zip › S1_Raw_data/Fig 2L.pdf]

WT-1

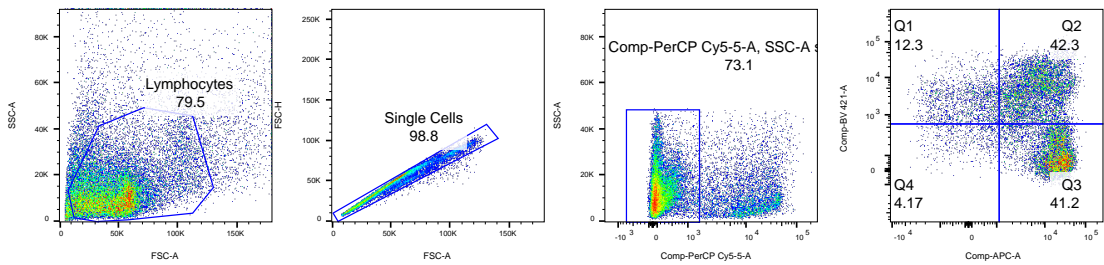

WT-2

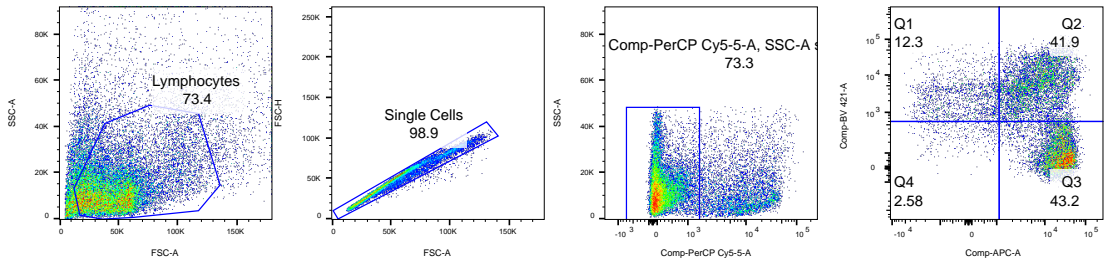

KO-1

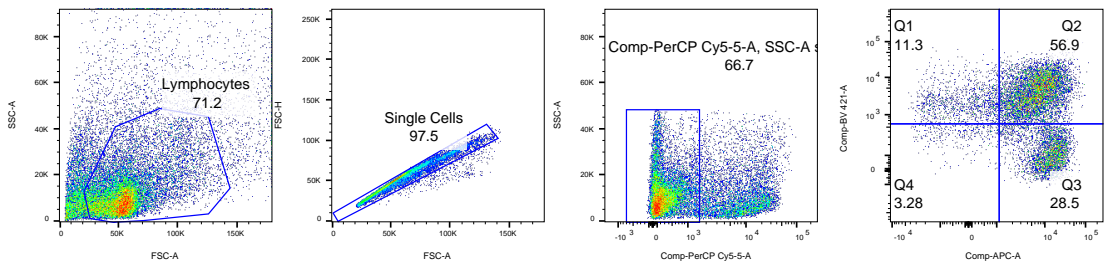

KO-2

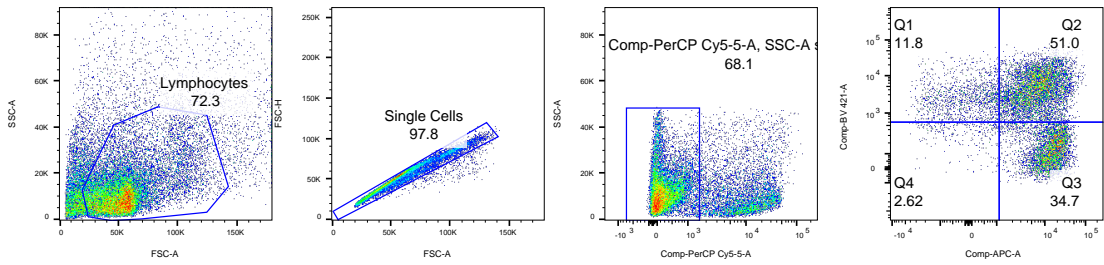

Supplement: S1 Raw data — (ZIP) [file pbio.3001811.s013.zip › S1_Raw_data/Fig 4B.pdf]
