# Supplementary material for: The inner nuclear membrane protein NEMP1 supports nuclear envelope openings and enucleation of erythroblasts
Source: PLoS Biol. 2022 Oct 10;20(10):e3001811. doi: 10.1371/journal.pbio.3001811 (PMC9595564; doi:10.1371/journal.pbio.3001811)
Supplement: S1 Text — (DOCX) [file pbio.3001811.s012.docx]

***S1 text: Nemp1* KO mice display increased splenic stress erythropoiesis in homeostatic conditions**

Because *Nemp1* KO mice have a higher number of stress-erythroid progenitors (Fig. 2L, M), we expected that *Nemp1* KO mice might recover relatively normally from acute anemia induced by phenylhydrazine (PHZ). Thus, WT and *Nemp1* KO mice were treated with PHZ and monitored for peripheral RBC recovery. As expected, *Nemp1* KO mice recovered similarly to WT mice from PHZ induced anemia (S1A Fig). We monitored peripheral RBCs after splenectomy to assess the splenic SEP contribution to ongoing erythropoiesis. We found that RBCs, Hb, and HCTs in the periphery were greatly reduced in *Nemp1* KO mice compared to WT mice (S1B Fig). Mean corpuscular volume (MCV) and RDW measures were significantly increased in *Nemp1* KO mice (S1B Fig). When splenectomized WT and *Nemp1* KO mice were challenged with PHZ, *Nemp1* KO mice showed much reduced recovery of RBC, Hb, and HCT (S1C Fig). Collectively, these data suggest that *Nemp1* deficient mice have increased stress erythropoiesis occurring in the spleen.
